# Validation of the Chinese version of the "Mood Disorder Questionnaire" for screening bipolar disorder among patients with a current depressive episode

**DOI:** 10.1186/1471-244X-12-8

**Published:** 2012-01-31

**Authors:** Zhaoyu Gan, Zili Han, Kanglai Li, Feici Diao, Xiaoli Wu, Nianhong Guan, Jinbei Zhang

**Affiliations:** 1 Psychiatry Department, the Third Affiliated Hospital, Sun Yat-sen University, Guangzhou 510630, China; 2 VIP Department, the Third Affiliated Hospital, Sun Yat-sen University, Guangzhou 510630, China

## Abstract

**Background:**

The Mood Disorder Questionnaire (MDQ) is a well-recognized screening tool for bipolar disorder, but its Chinese version needs further validation. This study aims to measure the accuracy of the Chinese version of the MDQ as a screening instrument for bipolar disorder (BPD) in a group of patients with a current major depressive episode.

**Methods:**

142 consecutive patients with an initial DSM-IV-TR diagnosis of a major depressive episode were screened for BPD using the Chinese translation of the MDQ and followed up for one year. The final diagnosis, determined by a special committee consisting of three trained senior psychiatrists, was used as a 'gold standard' and ROC was plotted to evaluate the performance of the MDQ. The optimal cut-off was chosen by maximizing the Younden's index.

**Results:**

Of the 142 patients, 122 (85.9%) finished the one year follow-up. On the basis of a semi-structured clinical interview 48.4% (59/122) received a diagnosis of unipolar depression (UPD), 36.9% (45/122) BPDII and 14.8% (18/122) BPDI. At the end of the one year follow-up,9 moved from UPD to BPD, 2 from BPDII to UPD, 1 from BPDII to BPDI, the overall rate of initial misdiagnosis was 16.4%. MDQ showed a good accuracy for BPD: the optimal cut-off was 4, with a sensitivity of 0.72 and a specificity of 0.73. When BPDII and BPDI were calculated independently, the optimal cut-off for BPDII was 4, with a sensitivity of 0.70 and a specificity of 0.73; while the optimal cut-off for BPDI was 5, with a sensitivity of 0.67 and a specificity of 0.86.

**Conclusions:**

Our results show that the Chinese version of MDQ is a valid tool for screening BPD in a group of patients with current depressive episode on the Chinese mainland.

## Background

Many studies have reported that patients with BPD are frequently misdiagnosed with other disorders. The frequency of initial misdiagnosis was reported to be as high as 69%, with more than one third of patients with BPD incorrectly diagnosed for up to ten years or longer [1]. At the same time, over-diagnosis of BPD is also reported to be common. Previous studies [2,3] showed that the frequency of over-diagnosis could be more than 50%. Inaccurate and delayed diagnosis can often lead to inappropriate treatment, which in turn results in poor outcome [4].

A number of strategies have been proposed to improve the detection of BPD in clinical practice. Using the Mood Disorder Questionnaire (MDQ) [5] is one of the common strategies. MDQ has been translated into many languages and has been proved to be a helpful tool in screening BPD [6-9]. A Chinese version of MDQ (Additional file 1: Chinese version of Mood Disorder Questionnaire) is a useful screening tool for BPD in a psychiatric population but not in the general population in Hong Kong [9,10]. However, the psychometric properties of MDQ were found to differ slightly under different language settings and among different populations [6-9].

The MDQ's poor performance in identifying mild bipolar spectrum, such as BPDII [11], greatly reduces its value, since BPDII accounts for the majority of misdiagnosis among patients with BPD in clinical practice [1,12]. Section 2 and 3 of MDQ might partly contribute to this problem [11,13,14]. Another potential reason might be the gold standard used as a reference. Over the past decades, most studies used a single structural clinical interview as a gold standard for diagnostic evaluation. However, a number of studies have shown that a single structural clinical interview based on DSM-IV criteria is far from enough to achieve an accurate diagnosis of BPD in clinical practice, especially for those with BPDII [15]. For instance, according to the variation of observation length, approximate 12.5%-30% patients with an initial diagnosis of UPD eventually received a diagnosis of BPD [16-18].

In the present study, we hypothesized that, among patients with current depressive episode, it might be reasonable and valuable to use MDQ as a screening tool for BPD, since most of those patients with bipolar spectrum disorder, especially BPDII, visit doctors when they are depressed, which makes them more likely to be misdiagnosed as UPD [17]. We decided to follow up patients for one year to evaluate the initial diagnosis based on a SCID-I interview in an attempt to improve the accuracy of the gold standard. Finally, the performance of MDQ without section 2 and 3 was also assessed.

## Methods

### Subjects

This study sample consisted of 142 eligible subjects that were treated currently for major depressive episode (MDE) based on the criteria of DSM-IV-TR in the psychiatric department, the 3^rd ^Affiliated Hospital of Sun Yat-sen University between July 2006 and July 2007. Written informed consent was obtained from all participants, and all procedures used in the present study were reviewed and approved by the local institutional review board. Patients with a psychiatric or physical disorder that prevented them from being interviewed or undermined their ability to provide accurate information, and those who declined participation in the study or refused to provide informed consent were excluded.

### Instruments

The Chinese version of SCID-I [19]: Chinese version of the Structured Clinical Interview for the Diagnostic and Statistical Manual of Mental Disorders, Fourth Edition, Text Revision (DSM-IV-TR) Axis 1 Disorders (SCID-I) was used for diagnostic interview.

MDQ [5]: The translation of MDQ into Chinese was approved by one of the developers of the original version. The Chinese version was translated back into English and re-edited to make it comparable to the original version.

### Procedure

Prior to the start of this study, three senior psychiatrists (HZL, GNH and WXL) attended a training program focused on SCID-I. At the end of the program, their inter-rater reliability was high, with a kappa coefficient of 0.93. Throughout the study period, all diagnostic interview and assessments were performed by these three psychiatrists, who constituted a special committee responsible for these tasks and were blind to the result of the MDQ.

Potential participants for this study were found by a study nurse (LKL) through reviewing the archive records and clinical outpatient files. The cases were included if they had been or would like to be followed up by the psychiatrists of our department. At the study entry, participants were invited (by LKL) to fill in the Chinese version of MDQ. SCID-I was performed for each participant to establish an initial diagnosis meeting the criteria of DSM-IV-TR. Demographic and clinical characteristics and features of the current depressive episode were collected using the self-compiled questionnaire. The participants were then followed up for one year, being interviewed by one of the three senior psychiatrists for at least six times with a flexible interval of 1-2 months via telephone or face to face. At each interview, if suspected diagnostic change was detected, the patient's relatives or friends were asked to provide additional information and the patient was asked whether they had similar experience before. All the data about the patient was then submitted to the committee, who would decide whether the patient had experienced a change in diagnosis or had experienced an earlier unrecognized manic or hypomanic episode, according to the criteria of DSM-IV-TR. To insure the quality and objectivity of switch detection, those who did not complete the one year follow-up or who were not contacted for more than 6 times within the year were excluded. At the end of study, the committee reviewed the one year medical records and came up with a final diagnosis.

During the study period, all treatment decisions or changes in treatment medications such as dose reduction, dose augmentation, or switch strategies were made by their treating psychiatrists. This study was carried out under naturalistic clinical settings and no treatment information was obtained.

### Statistical analysis

All statistical analysis was performed using commercial statistical package SPSS 13.0(SPSS Inc., Chicago). The Mann-Whitney *U *test was used to compare numerical variables and the chi-square test was used to compare categorical variables. Cronbach alpha was used to access the internal consistency of the scale. The receiver operating characteristic (ROC) curve was plotted to assess the screening performance of the questionnaire. Its accuracy was calculated in terms of sensibility and specificity for each theoretically possible cut-off, and then the method of linear interpolation was used to calculate the sensibility and specificity for each actually possible cut-off (number of positive answers). The optimal cut-off was determined by maximizing the Youden's index (= sensitivity + specificity-1).

## Results

### Comparison of the dropout group and the rest

At the beginning of the study, 102 subjects (71.8%) were inpatients and 40 (28.2%) were outpatients. Of the 142 subjects, 122 (85.9%) completed the one year follow-up receiving 6-12(7 ± 2) visits. Reasons for dropout included transferring to another psychiatric institution (9 subjects, 6.33%) and refusal to continue the study (11 subjects, 7.74%). No difference was found between the dropout group and the rest with regard to demographic and clinical features. Therefore, patients who dropped out were excluded from subsequent analysis.

### Comparison of BPD and UPD with regard to demographic and clinical features

As Table 1 demonstrated, patients with BPD, compared to patients with UPD, were younger, had an earlier age onset, experienced a longer illness course and a larger number of depressive episodes, and more likely saw manic symptoms during a depressive episode. Patients with BPDI differed from patients with BPDII in illness course and the proportion of recurrent depression: Compared to patients with UPD, the former consisted of higher percentage of patients with recurrent depression while the latter experienced a longer illness course.

**Table 1:** The demographic and clinical features of the sample population

Features	BPD^a^			UPD	*P*^b^		
			
	BPDI	BPDII	Total		BPDI	BPDII	BPD
	**N = 20**	**N = 50**	**N = 70**	**N = 52**			

Age(Mean ± SD) (year)	28.0 ± 8.7	29.2 ± 8.6	28.8 ± 8.6	33.6 ± 10.5	0.023	0.009	0.007

Onset age (Mean ± SD) (year)	25.4 ± 8.2	25.7 ± 9.3	25.6 ± 8.9	31.2 ± 10.9	0.034	0.009	0.003

Duration of illness (month)	42.1 ± 34.4	51.8 ± 61.3	48.9 ± 54.6	30.8 ± 33.0	0.202	0.034	0.037

Female (%)	5(25.0)	27(54.0)	32(45.7)	29(55.8)	0.019	0.858	0.272

Atypical features^c ^(%)	6(30.0)	8(16.0)	14(20.0)	6(11.5)	0.060	0.513	0.212

Manic symptoms^d^(%)	6(30.0)	16(32.0)	22(31.4)	4(7.7)	0.014	0.002	0.002

Comorbidity of anxiety disorder^e ^(%)	7(35.0)	14(28.0)	21(30.0)	16(30.8)	0.730	0.759	0.927

Comorbidity of psychoactive drug abuse (%)	1(5.0)	1(2.0)	2(2.9)	3(5.8)	0.898	0.327	0.422

Recurrent depression (%)	15(75.0)	27(54.0)	42(60.0)	23(44.2)	0.019	0.324	0.084

Number of depressive episode(χ ± s)	3.6 ± 4.2	3.3 ± 4.4	3.4 ± 4.3	1.8 ± 1.0	0.006	0.015	0.009

a. The categorization was based on the final diagnosis

b. all compared with UPD

c. Atypical features including mood reactivity, overeating or weight gain, oversleeping, leaden paralysis and interpersonal rejection sensitivity

d. Manic symptoms, including speech pressure, racing thought, overconfidence, hostility, irritability, sexual interest and garish make-up were found by psychiatric interview based on the Young Mania Rating Scale at the entry of this study

e. Anxiety disorder here consisted of generalized anxiety disorder, panic disorder, obsessive-compulsive disorder, phobia, somatization disorder

### Comparison of initial diagnosis and final diagnosis

According to Table 2, both underdiagnosis and overdiagnosis of BPD existed in this study, but the underdiagnosis was commoner than overdiagnosis. Among the nine patients whose diagnosis switched from UPD to BPD, three were confirmed to have an undeclared hypomanic episode before entry into the study by other sources of information acquired during the follow-up visit, diagnosis was changed in six cases because of newly occurred manic or hypomanic episode during the one year follow-up (all admitted they had similar experience before). As for the two participants who were initially diagnosed as BPD but finally moved to UPD, they both rejected the treatment of mood stabilizers prescribed by their physicians because of poor tolerance, no switch was detected throughout the whole follow-up period though antidepressant treatment had been maintained. Reassessment of past data confirmed no clinically significant hypomanic episode had happened before.

**Table 2:** Comparison of initial diagnosis and final diagnosis

Initial diagnosis	Final diagnosis	N of cases	Percentage (%)
UPD(59)	UPD	50	84.7

	BPDII	8	13.6

	BPDI	1	1.6

BPDII(45)	UPD	2	4.4

	BPDII	42	93.3

	BPDI	1	2.2

BPDI(18)	UPD	0	0

	BPDII	0	0

	BPDI	18	100%

Total(122)	Agreed with initial diagnosis	110	83.6%
	
	Disagreed with initial diagnosis	12	16.4%

### The internal consistency of the Chinese version of the MDQ

In this sample, the Cronbach coefficient for the 13-item symptom scale was 0.735, the item-total scale correlation ranged from 0.195 (less sleep) to 0.597 (more active). The elimination of each item did not greatly alter the scale's internal consistency.

### Scores of MDQ in each section

Table 3 showed summary scores of all subjects in each section of MDQ. The high proportion (34.7%) of subjects with UPD scoring moderate or severe in section 3 and the high prevalence of missing value in the section 2 and 3 indicated that there might be some misunderstanding in these sections. While the percentage of those scoring moderate or severe among subjects with BPD was less than expected, if combined with the number of symptoms, this percentage would be lower. Therefore, section 2 and 3 make no sense in distinguishing BPD from UPD and were excluded from subsequent analysis.

**Table 3:** Scores of the subjects in each section of MDQ

MDQ		BPDI^a^	BPDII^a^	UPD^a^
		(N = 20)	(N = 50)	(N = 52)
**Section 1(**Number of positive answers)	$\bar{x}\text{±}s$	6.85 ± 3.33	5.76 ± 2.73	3.02 ± 2.00**^b^

**Section2**	Yes	12(60.0%)	26(50%)	12(23.1%)
	
	No	6(30.0%)	16(34.6%)	30(57.7%)
	
	Missing	2(10.0%)	8(15.4%)	10(19.2%)

**Section 3**	unaffected	4(21.1%)	6(12.0%)	10(19.2%)
	
	mild	7(36.8%)	12(24.0%)	14(26.9%)
	
	moderate	3(15.8%)	16(32.0%)	11(21.2%)
	
	severe	3(15.8%)	13(26.0%)	7(13.5%)
	
	missing	2(10.5%)	3(6.0%)	10(19.2%)

a. the final diagnosis after one year follow-up

b. compared to subjects with BPD, subjects with UPD scored significantly lower (*P *< 0.01) in section 1 of MDQ

### ROC analysis of section 1

ROC was plotted according to the scores obtained in section 1. The corresponding sensibility and specificity for each possible cut-off (number of positive answers) were calculated by linear interpolation based on the sensibility and specificity of the corresponding theoretical cut-off in ROC. The results and the corresponding area under curve (AUC) and p value were listed in Table 4. By maximizing the Youden's index, 4 was selected as the optimal cut-off for patients with BPD or BPDII, with a sensibility of 0.72 or 070 respectively and a specificity of 0.73. If BPDI was separately calculated, 5 was considered the optimal cut-off, with a sensibility of 0.67 and a specificity of 0.86.

**Table 4:** ROC analysis of section1 for BPD, BPDII and BPDI

	AUC	*P*	Cut-off	1	2	3	4	5	6	7	8	9	10	11	12
BPD	0.803	< 0.001	sen	0.98	0.93	0.85	**0.72**	0.59	0.42	0.35	0.26	0.19	0.14	0.06	0.02
			
			spe	0.15	0.36	0.55	**0.73**	0.86	0.90	0.94	0.97	0.99	1.00	1.00	1.00

BPDII	0.794	< 0.001	sen	0.99	0.94	0.84	**0.70**	0.55	0.43	0.31	0.20	0.13	0.09	0.05	0.03
			
			spe	0.15	0.36	0.55	**0.73**	0.86	0.90	0.94	0.97	0.99	1.00	1.00	1.00

BPDI	0.826	< 0.001	sen	0.95	0.90	0.88	0.78	**0.67**	0.58	0.50	0.43	0.33	0.25	0.13	0.00
			
			spe	0.15	0.36	0.55	0.73	**0.86**	0.90	0.94	0.97	0.99	1.00	1.00	1.00

**a**. sen = sensibility, spe = specificity

**b**. The sensibility and specificity were calculated by linear interlocation based on the sensibility and specificity of the corresponding cut-off on ROC

**c**. the sensibility and specificity in boldface maximized the Youden's index and the corresponding cut-off was considered the optimal one

### Scores of section 1 among participants with diagnosis changed during the follow-up

Compared to 50 subjects who maintained the diagnosis of UPD, subjects whose diagnosis changed from UPD to BPD scored significantly higher in section 1 of the MDQ (2.7 ± 1.7 vs. 5.2 ± 3.0, *p *= 0.036).

## Discussion and Conclusions

As this study and our previous report [20] have shown, the diagnosis of UPD and BPD based on a single interview is unstable over time, with 16.4% to 19.4% of subjects changing diagnosis, similar to the range of 11.7% to 19.7% reported in other studies [16,21,22]. Using a diagnosis obtained at one year follow-up as 'gold standard' helps obtain a more reliable assessment.

Compared to the optimal cut-off of 7 reported by studies from western countries [5,7] and Hong Kong [9], this study showed a smaller optimal cut-off, which was similar to findings from Chinese mainland [23]. This might partly due to the cultural differences, since Hong Kong is a very westernized city in China, which makes its culture and language greatly different from Chinese mainland.

In line with previous studies [5,7,8], the MDQ is more sensitive in detecting BPDI than detecting BPDII. Although the originator of the MDQ did not specially access patients in remission from a mood episode, whether the patient's symptomatology at the time of screening will affect the MDQ performance is an interesting topic. A previous study with a small sample size [24] showed the performance of MDQ was independent of depressive symptoms, but the relatively low test-retest reliability(kappa coefficient 0.64) with the whole sample implicated the possible influence of clinically relevant factors, such as the patient's mood state at time of completion. While compared to a report which sampled patients treated for depression [15], the performance of the MDQ in detecting BPDII in this study was quite close (sensibility: 0.706 vs. 0.70), in spite of the different cut-off (7 vs. 4).

According to the initial conception of the MDQ's developers, a subject who will be screened positive has to meet the DSM-IV-TR criteria of manic or hypomanic episode, including symptom criteria and severity criteria. However, the poor performance of the section 2 and 3 in this study and other reports [6,7,15] indicates an inadequacy in the original conception, especially when screening patients with BPDII. In this study, we went further by adding a question to ask subjects how long the positive symptoms lasted. We found that 16(32%) subjects with BPDII did not meet the DSM-IV-TR duration criteria of hypomanic episode (lasting at least 4 days). That means it is unrealistic to expect a self-rated questionnaire to help improve recognition of a past hypomanic episode among patients with BPDII.

However, MDQ without section 2 and 3 has been shown to be a valid screening tool for BPDII, and even for previously unrecognized bipolar disorder [25]. One explanation for this might be that MDQ without section 2 and 3 helps recognize the opposite polarity-manic or hypomanic symptoms of BPDII, which helps improve the recognition of BPD [26,27]. Recently, convergent evidence has shown that bipolarity is a sensitive and characteristic feature of BPD [26,28,29]. For instance, a cross-sectional study [30] found that clinically significant depressive symptoms occurred in 94.1% of those with (hypo) mania, while 70.1% in a depressive episode had clinically significant manic symptoms. In addition, both prospective [31] and cross-sectional survey [32] found that major depressive disorder (MDD) with subthreshold bipolarity shared similarities with BPD and more likely converted into BPD during follow-up. In this study, manic symptoms were also found to be more likely to occur in patients with BPD than those with UPD. In this context, it is not difficult to understand why MDQ without section 2 and 3 can be used as a screening tool to detect bipolar diathesis in depression [28,33].

In summary, out study shows that the Chinese version of the MDQ without section 2 and 3 is a valid, brief and feasible tool for screening BPD from patients with a current depressive episode in Chinese mainland, although the psychometric properties in terms of internal consistency is not as excellent as reports in western countries [6,7], which means some modification is needed. Furthermore, the small sample size in our study makes a larger prospective study necessary to further testify the validation of the Chinese version of MDQ under different clinical settings.

## Competing interests

The authors declare that they have no competing interests.

## Authors' contributions

ZJB designed and organized the study, trained counselors conducting structured interview and supervised the quality of research. GZY designed the study, translated the MDQ and drafted the manuscript. HZL, as the chief of the special committee, conducted structured interview, collected other clinical information, followed up each subject and made final diagnostic decision. LKL recruited subjects, surveyed with MDQ, arranged follow-up visits and entered data. DFC translated the MDQ and conducted statistical analysis. WXL and GNH, both as member of the special committee, conducted structured interview, followed up each subject and made final diagnostic decision. All authors read and approved the final manuscript.

## Pre-publication history

The pre-publication history for this paper can be accessed here:

http://www.biomedcentral.com/1471-244X/12/8/prepub

## Supplementary Material

Additional file 1 **Chinese version of Mood Disorder Questionnaire**. Click here for file
