# Supplementary material for: Validation of the Chinese version of the "Mood Disorder Questionnaire" for screening bipolar disorder among patients with a current depressive episode
Source: BMC Psychiatry. 2012 Jan 31;12:8. doi: 10.1186/1471-244X-12-8 (PMC3299660; doi:10.1186/1471-244X-12-8)
Supplement: Additional file 1 — Chinese version of Mood Disorder Questionnaire. [file 1471-244X-12-8-S1.DOC]

**心境障碍调查问卷**

**提示语:**仔细阅读以下题目，选择一个最符合您实际情况的答案。

1. **您是否曾经有一段时间感觉自己跟平时不一样，例如… 是 否**

感觉心情特别好或非常兴奋以致别人觉得您有点不正常或者由于太兴奋导致您惹来麻烦?――― □ □

变得非常易激惹以致对别人大声喊叫甚至争吵或者打架? ―――――――――――――――――□ □

感觉到比以往更自信? ― ―――――――――――――――――――――――――――――― □ □

.睡眠时间比平时明显减少却没有睡不够的感觉?―――――――――――――――――――― □ □

比平时更加健谈而且讲话的速度比平时快?―――――――――――――――――――――――□ □

思维变得十分敏捷，感觉无法将自己的思维慢下来?―――――――――――――――――― □ □

.注意力容易受外界的干扰以致无法集中或专注于某一件事情上?――――――――――――― □ □

.精力比平时更加充沛?――――――――――――――――――――――――――――――― □ □

.比平时更加活跃或做更多的事情，如变得乐于助人、爱管闲事?――――――――――――― □ □

.社交或户外活动明显增多，如半夜打电话与朋友聊天?――――――――――――――――― □ □

性欲比平时增强，对异性特别感兴趣?―――――――――――――――――――――――― □ □

.做一些对您来说不寻常的或别人认为是多余的、愚蠢的或冒险的事情?―――――――――― □ □

挥霍无度、花钱大手大脚以致使自己或家庭陷入经济困境?――――――――――――――― □ □

**2. 如果您在上述题目中有多条题目选择了是, 请问这些曾经发生过的事情是否是在同一时间内同时发生?**

**是**  **否**

**3. 上述曾经发生的事情对您的影响有多大 – 如不能上班; 招致家庭矛盾、经济困境或官司缠身;争吵或斗殴、人际关系紧张? 请选择以下最适合您的其中一项**
无影响 轻微的影响中等程度的影响  严重的影响
